# Functional roles of the hexamer structure of C‐phycocyanin revealed by calculation of absorption wavelength

**DOI:** 10.1002/2211-5463.13038

**Published:** 2020-11-29

**Authors:** Hiroto Kikuchi

**Affiliations:** ^1^ Department of Physics Nippon Medical School Musashino Japan

**Keywords:** absorption wavelength, C‐phycocyanin, energy transfer, phycobilisome, phycocyanobilin, structure and function

## Abstract

Cyanophyta‐phycocyanin (C‐PC) is the main constituent of the rod of phycobilisome (PBS), which is a highly ordered and large peripheral light‐harvesting protein complex present on the cytoplasmic side of the thylakoid membrane in cyanobacteria and red algae. The C‐PC monomer comprises two chains, α‐ and β‐subunits, and aggregates to form ring‐shaped trimers (αβ)_3_ with rotational symmetry. The ring‐shaped trimer (αβ)_3_ is a structural block unit (SBU) that forms the rod of PBS. Two (αβ)_3_ SBUs are arranged in a face‐to‐face manner to form an (αβ)_6_‐hexamer. In this study, the electronic states of three phycocyanobilins, α84, β84, and β155 in C‐phycocyanin, constituting the rod of the PBS, were calculated for both the trimer and hexamer models by considering the effect of the electrostatic field of protein moieties and water molecules. For the hexamer, the absorption wavelengths of α84, β84, and β155 were similar to those obtained experimentally; however, for the trimer, only the absorption wavelength of β155 shifted toward a shorter‐wavelength. The nature of the hexamer structure as a hierarchical structure is revealed by considering the calculated absorption wavelength and energy transfer.

## Graphical abstract

The phycocyanin trimer structure is the basic building block of the rod of phycobilisome (PBS). However, PBS is composed of one higher hierarchical structure, a unit of hexamer structure that combines two trimers. In this study, we clarify the functional role of the hexamer structure from the viewpoint of light absorption properties and energy transfer of chromophore β155.

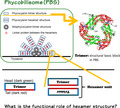

**Abbreviations**
C‐PC: C‐phycocyanine
INDO‐CI: Intermediate Neglect of Differential Overlap‐Configuration Interaction
MO: molecular orbital
PBS: phycobilisome
PCB: phycocyanobilin
S_0_→S_1_: the transition from the ground state to the first excited state

C‐phycocyanin (C‐PC) is the main constituent of the rod of phycobilisome (PBS), which is a highly ordered and large peripheral light‐harvesting protein complex occurring on the cytoplasmic side of the thylakoid membrane in cyanobacteria and red algae [1, 2, 3]. Herein, ‘C’ indicates the type of source organism (Cyanophyta). A schematic representation of PBS is shown in Fig. 1A. The C‐PC monomer comprises two chains, α‐ and β‐subunits, and aggregates to form ring‐shaped trimers (αβ)_3_ with rotational symmetry (Fig. 1B). In both the α‐ and β‐subunits, phycocyanobilin (PCB), that is, a chromophore, is bound to cysteine‐84 by a cysteinyl thioether linkage through the vinyl substituent on the pyrrole ring. The β‐subunit contains an additional PCB bound to cysteine‐155 in a manner similar to cysteine‐84. These PCBs are called α84, β84, and β155, respectively [4, 5].

**Fig. 1:**
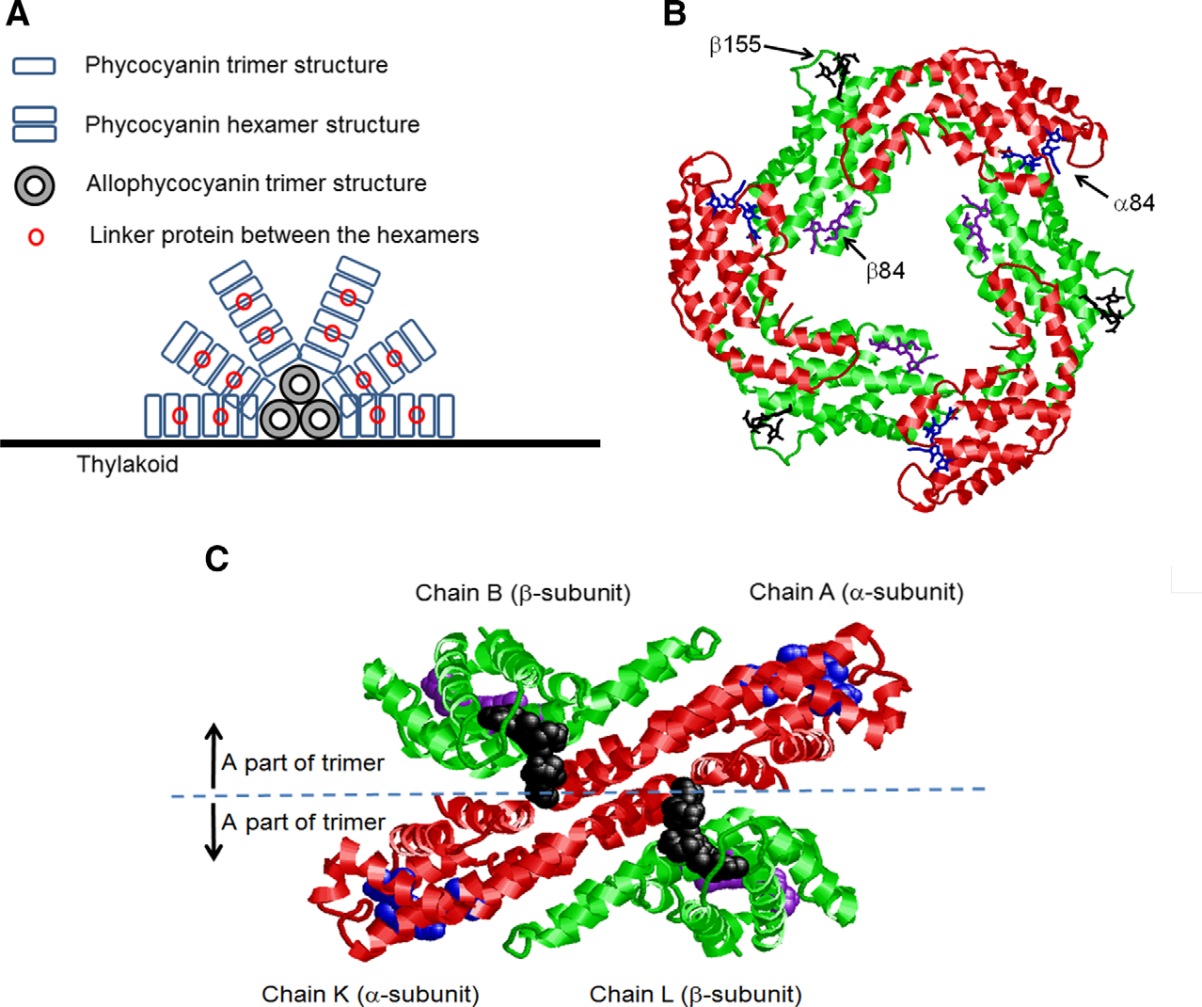
Schematic representation of PBS, three‐dimensional structures of the trimeric (αβ)_3_ C‐phycocyanin isolated from cyanobacterium *Fremyella diplosiphon* (Dürring *et al*. [13]; PDB ID, 1CPC), and a part of the hexamer in which two trimers are associated with each other. In (A), the trimer form that corresponds to (B) is depicted by the rectangle (dark blue), and the linker protein connecting the hexamers is depicted by the red circle. There are other linker proteins between allophycocyanins constituting the PBS core, between the rod and the core, and between the core and the thylakoid membrane; however, they are omitted in (A). The reaction center complexes are located within the membrane underneath the cores. In (B) and (C), the α‐subunit (red) and the β‐subunit (green) are drawn using the ribbon model. The chromophores, α84 (blue), β84 (purple), and β155 (black), are drawn by the stick model. In (C), it is shown how the α‐ and β‐subunits belonging to one trimer contact the α‐ and β‐subunits belonging to the other trimer.

The ring‐shaped trimer (αβ)_3_ is a structural block unit (SBU) that forms the rod of PBS. Two (αβ)_3_ SBUs are arranged in a face‐to‐face manner to form an (αβ)_6_‐hexamer. Two (αβ)_6_‐hexamers are associated in a back‐to‐back fashion via a linker protein [6]. Shirmer *et al*. [5] analyzed the crystal structure of hexameric C‐PC obtained from cyanobacterium *Agmenellum quadruplicatum* and showed that the (αβ)_6_‐hexamer is the functional unit of the native PBS. On the other hand, almost the same time, Mimuro *et al*. pointed out from experimental absorption spectra, CD, fluorescence, and fluorescence polarization spectroscopy that first of all the absorption energy of β155 transfers to β84 in the (αβ)_3_‐trimer, and thus, an (αβ)_3_‐trimer is not only a SBU but also the functional unit of the energy transfer [7].

Although the term ‘functional unit’ was used while focusing on the direction of energy flow, the pathway of energy flow is not the only function of PBS. For example, light adaptation or chromatic acclimation (CA) processes in PBS function in hexamer units [8, 9, 10]. If the hexamer is not a hierarchical structure with functional meaning, then light adaptation or CA processes in PBS might have functioned in trimeric units. To discuss the implications of the hexamer structure being one of the hierarchical structures of PBS from the functional view is inevitable in understanding the relationship between function and structure in PBS. Additionally, to consider the relationship between the hierarchical structure of PBS and its function could lead to understanding the diversity that makes PBS an amazing light‐harvesting system or the evolutionary issues of PBS [11, 12].

In this study, the electronic states of three chromophores were determined on the basis of the crystal structure [13], including the effects of peptide moieties and water molecules, of both the trimer and hexamer. The functional role of the hexamer structure is also investigated based on the results obtained.

## Materials and methods

C‐PC was isolated from the cyanobacterium *Fremyellia diplosiphone* [13]. The resolution of the crystal data was 1.66 Å in Protein Data Bank (PDB; PDB ID: 1CPC).

### Net charges and hydrogen coordinates of the α‐ and β‐subunits

Before calculating light absorption, the coordinates of the hydrogen atoms were determined. First, three successive amino acid residues in the α‐subunit or the β‐subunit of 1CPC were treated as a trimer, to which hydrogen atoms were added. Then, the coordinates of the hydrogen atoms were optimized with respect to the total energy of the trimer by using the Modified Neglect of Diatomic Overlap—Parametric Method 3 molecular orbital (MO) method [14, 15]. For the trimer, the N terminus was set to ‐NH_2_, but the C terminus, except for the C terminus of C‐PC, was replaced with ‐COCH_3_ because the oxygen atom in ‐OH strongly attracts electrons. At the C terminus of C‐PC, ‐COOH was used. The coordinates and net charges of the central amino acid residue in the trimer were calculated and used. These procedures were performed until the coordinates, and net charges of all the amino acid residues in C‐PC were obtained. These values were then utilized for determining the wavelength of light absorption.

### Coordinates and net charges in water molecules

Molecular dynamics simulations were performed using Amber 12 to determine the coordinates of water molecules around the trimer or hexamer of C‐PC [16]. The Amber ff03 force field [17, 18] was used for the proteins. A part of the hexamer comprising A(α‐subunit), B(β‐subunit), K(α‐subunit), and L(β subunit) in 1CPC (see Fig. 1C) or the trimer by itself was immersed in water molecules. Hereinafter, the former is called the hexamer model and the latter is called the trimer model. Both models were electrostatically neutralized by counter ions, and an explicit water box (TIP3P) was used. The systems were minimized for 300 steepest‐descent steps and equilibrated for 1 ns by gradually increasing the temperature. Finally, 100‐ns production runs were performed. The positions of the atoms, except for water molecules, were fixed during the calculation. The temperature and pressure were kept constant by using Berendsen rescaling methods [19], and long‐range electrostatic forces were computed using the particle‐mesh Ewald method [20]. The net charges of the oxygen and hydrogen atoms of water molecules were −0.3307 and 0.1653, respectively. These values were obtained by using the *ab initio* MO method for a water molecule with the gaussian09 software [21] (Gaussian, Inc., Wallingford, CT, USA) at the HF/ STO‐3G level.

### Calculation of the wavelength of light absorption and the oscillator strength

The wavelength of light absorption and the oscillator strength were calculated using the unique intermediate neglect of differential overlap‐configuration interaction (INDO‐CI) method [22]. All molecular integrals in the calculation were estimated as functions of electron densities of individual atoms according to Sakuranaga *et al*. [22]; this INDO‐CI method is slightly different from that used by Pople *et al*. [23]. The resonance integrals were expressed using parameter *k*_β_ in equation (2.2) in ref. 22, as described by Wolfsberg‐Helmholtz [24]. In the present study, all the values except for that of *k*_β_ are the same as those reported by Sakuranaga *et al*. [22]. The values of *k*_β_ were *k*_β_(C‐C) = *k*_β_(C‐O) = 1.10, *k*_β_(C‐N) = 0.70, and *k*_β_(others) = 1.20 to reproduce the observed light absorption and oscillator strength of the individual bands of the α84 chromophore in C‐PC [25]. Each chromophore was treated as a protonated form [25, 26, 27, 28, 29].

A total of 338 lowest singly excited configurations, ψ (*j*, *m*), and doubly excited configurations, ψ (*jj*, *mm*), were considered for calculating the CI. Herein, ψ (*j*, *m*) was constructed by exciting an electron from an occupied MO ϕ*_j_* to an unoccupied MO ϕ*_m_*, and ψ (*jj*, *mm*) was constructed by exciting a pair of electrons from ϕ*_j_* to ϕ*_m_*. For CI calculations, the interaction between the chromophore and its surrounding protein moieties or water molecules was considered as the electrostatic interaction between the electronic states of the chromophore and the net charges of its surrounding atoms.

**Fig. 2:**
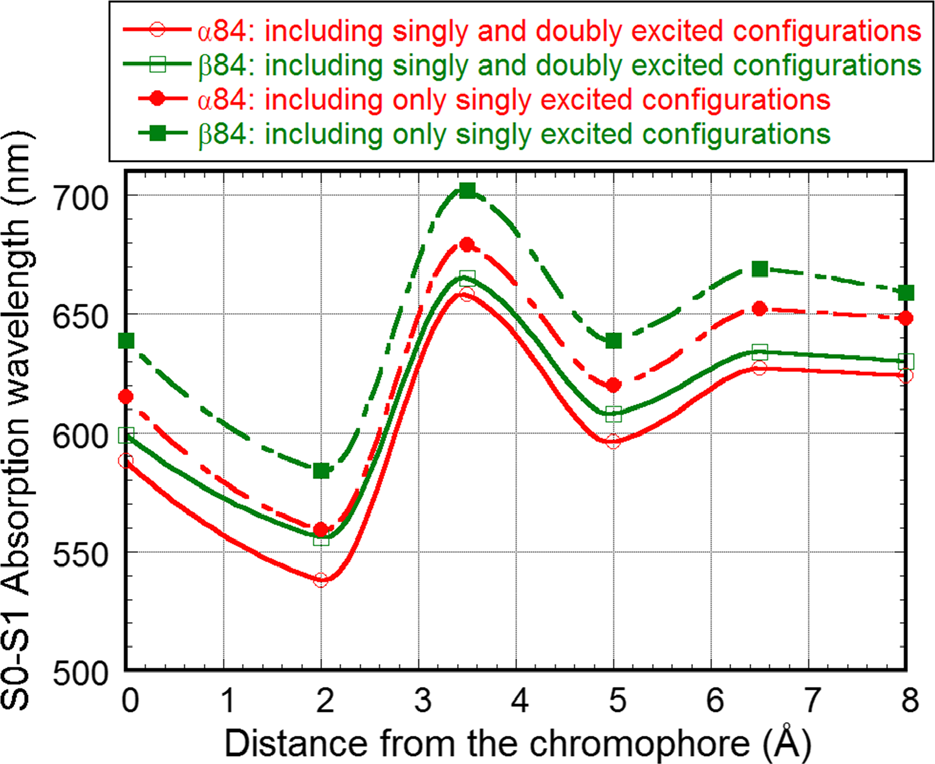
Dependence of λ_1_ for the S_0_→S_1_ transition on distance *R* from the α84 or β84 chromophore, calculated by including the electrostatic interaction of the α84 or β84 chromophore with protein moieties and water molecules within *R* Å. *R* from the chromophore means the longest distance from a given atom of the chromophore to any atom of the protein or water.

Additionally, for comparison, the calculations using only 169 lowest singly excited configurations, ψ (*j*, *m*), for the CI were carried out for α84 and β84. Figure 2 shows the dependence of λ_1_ for the S_0_→S_1_ transition on distance *R* from a given atom of the α84 or β84 chromophore to any atom of the protein moiety and water molecule when the number of only the lowest singly excited configurations, ψ (*j*, *m*), and the lowest singly and doubly excited ones, ψ (*j*, *m*) and ψ (*jj*, *mm*), were 169 and 338, respectively. The wavelength λ_1_ obtained for the S_0_→S_1_ transition with only the single‐CI was about 5% longer than that obtained using the single‐CI and double‐CI with excitations of two electrons from the same orbital. However, the tendency of the distance dependence was the same for both α84 and β84 (Fig. 2).

Even if the larger set of excitations between two different orbitals, ψ (*kj*, *mn*), for the CI calculations were considered, the tendency of the distance dependence would show the same tendency including the slight difference in the absorption wavelength. And this difference can be regulated by the values of parameters *k*_β_. Indeed, it is important for more accurate results to consider the larger set of excitations between two different orbitals, ψ (*kj*, *mn*), for the CI calculations. However, to validate the purpose of the present study, it is sufficient to consider the single‐CI and double‐CI containing only excitations of two electrons from the same orbital. Thus, in this study, the values of parameter *k*_β_ were set based on this level of CI calculations.

## Results

Mimuro *et al*. reported [7] that the maximum light absorption of α84, β84, and β155 is obtained for the transition from the ground state to the first excited state (S_0_→S_1_), and the wavelength for each transition is 618, 625, and 594 nm, respectively. Almost the same value has been reported elsewhere too. When the chromophore is electrically neutral, the calculated oscillator strength *f*_1_ for the transition (S_0_→S_1_) is small and does not agree with the experimental result. However, when the chromophore is protonated, namely when each nitrogen atom of the central pyrrole rings B and C combines with a hydrogen atom, the calculated oscillator strength *f*_1_ for S_0_→S_1_ increases and agrees with the experimental result. In this study, the protonated form was used and λ_1_ (λ_max_), which is the absorption wavelength for S_0_→S_1_, was utilized as an index.

The chemical geometry of a chromophore is the chief factor that determines its electronic state, followed by its protonation, and the subsequent effect is the electrical interaction from the environment, which consists of the atoms of amino acid in the protein moiety and water molecules. Since the electrical effect of the environment was considered to be distance‐dependent, the effect on the electronic state of the chromophore from the environment was estimated by using constancy of the λ_1_ of the S_0_→S_1_ transition as a measure. When the atoms of amino acids and water molecules within 7 or 8 Å from α84 or β84 for the trimer model were taken into account, the fluctuations of λ_1_ suppressed (Fig. 2).

**Fig. 3:**
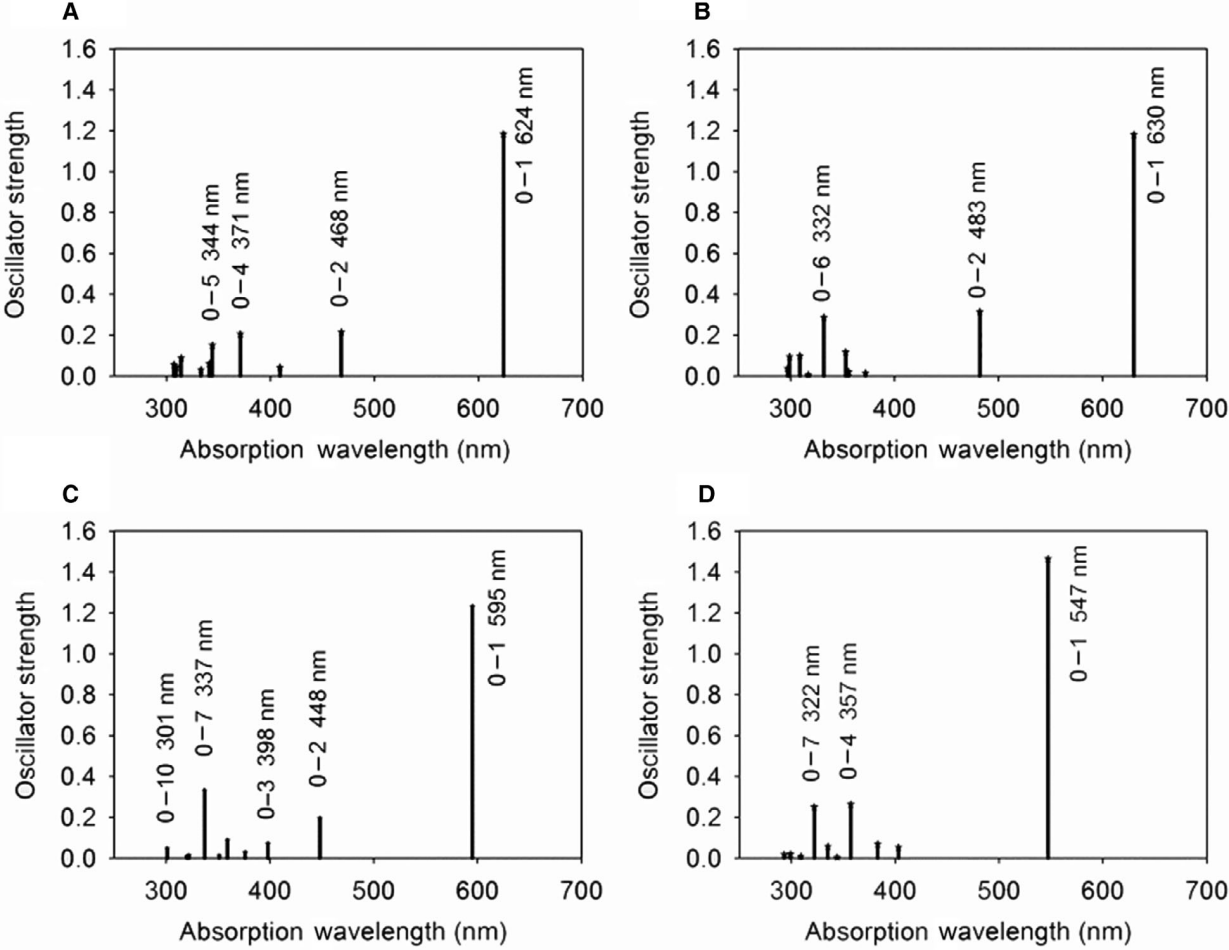
Optical absorptions of chromophores of C‐PC on a scale of oscillator strength versus wavelength, calculated for the transition from the ground state to the *r*th excited states by using the INDO‐CI method of Sakuranaga *et al*. [22]: (A) α84; (B) β84; (C) β155 of the hexamer model; (D) β155 of the trimer model.

Figure 3A,B show the calculated absorption wavelength of α84 and β84 with the protein moieties and water molecules within 8 Å from the α84 and the β84, respectively. For α84, λ_1_ is 624 nm and *f*_1_ is 1.18, whereas for β84, λ_1_ is 630 nm and *f*_1_ is 1.18. The position and orientation of the water molecule in case of α84 are different from those used through the previous calculation [25], but almost the same wavelength as the previous calculation result was obtained. This implies that α84 is present inside the protein and is largely unaffected by the water outside the PBS. In this study, the calculation result of β84 was also obtained, and the λ_1_ of β84 was slightly longer than that of α84. This result almost reproduces the experimental observations [7, 30]. Although the interaction with the linker protein was not taken into account, this effect would be more helpful for understanding the energy transition mechanism in PBS.

The surrounding environment for α84 or β84 of the hexamer model is the same as that of the trimer model when they are considered within 8 Å from the chromophore. However, for β155, a difference in the surrounding environment between the trimer and hexamer models was observed. Figure 3C,D show the calculated results of the trimer and hexamer models, including the condition when the protein moieties and water molecules are within 8 Å from β155. For the hexamer model (Fig. 3C), the calculation result (λ_1_ = 595 nm and *f*_1_ = 1.23) was the same as the experimental result for native PBS; however, for the trimer model (Fig. 3D), λ_1_ shifted to the shorter‐wavelength side (λ_1_ = 547 nm and *f*_1_ = 1.46) than the experimental value (594 nm [7]). In other words, the calculated absorption wavelengths of α84, β84, and β155 reproduced the experimental results [7, 30, 31] in the case of the hexamer. In contrast, for the trimer, the wavelength of β155 shifted to the shorter‐wavelength side.

**Fig. 4:**
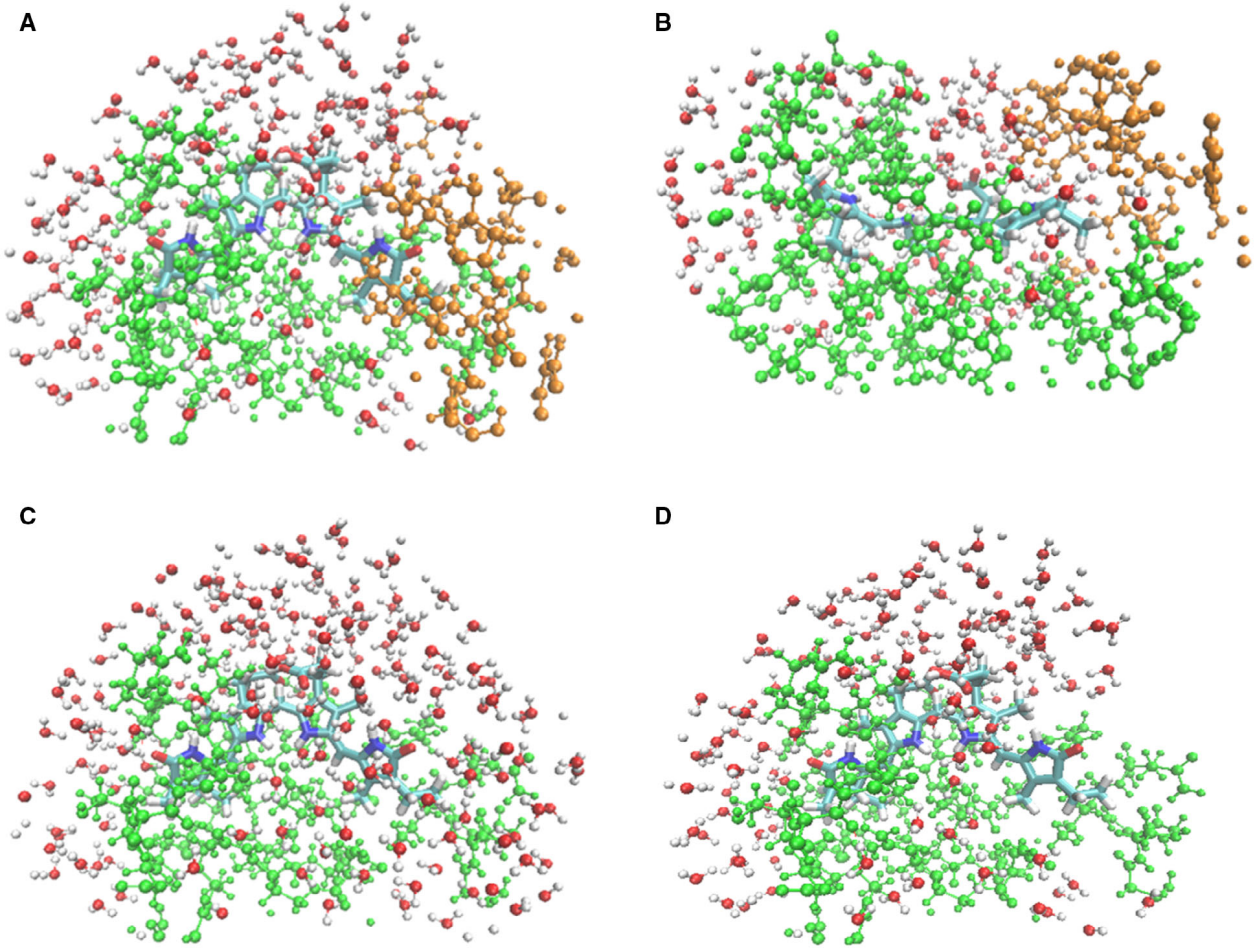
β155 and the atoms of protein moieties and water molecules within 8 Å from β155: (A) the hexamer model; (B) the hexamer model viewed from different angles from (A); (C) the trimer model; (D) the model after removing the K chain part from the hexamer model, namely from (A). β155 is drawn using the licorice model, and the atoms belonging to the trimer part (green) and the atoms belonging to the K chain (orange) are drawn by the ball‐and‐stick model. The oxygen (red) and the hydrogen atom (white) of the water molecule are also drawn by the ball‐and‐stick model. Then, figures (A), (C), and (D) are viewed from the same angle.

Figure 4A,B show the atoms within 8 Å from β155 considered in the hexamer model. The chromophore is depicted in a licorice representation, and the atoms belonging to the trimer model and the other atoms that belong to the α‐subunit of the adjacent trimer (K chain in 1CPC) are represented by green and orange, respectively. As shown in these figures, β155 interacts with the K chain, which is included in the hexamer model, but not in the trimer model. This can also be seen in Fig. 1C.

Figure 4C shows the atoms within 8 Å from β155 considered in the trimer model, and Fig. 4D shows the model by removing the K chain from the hexamer model. For the trimer model, most of the periphery of β155 is covered with water molecules, which is a different situation from that of the native PBS environment.

**Fig. 5:**
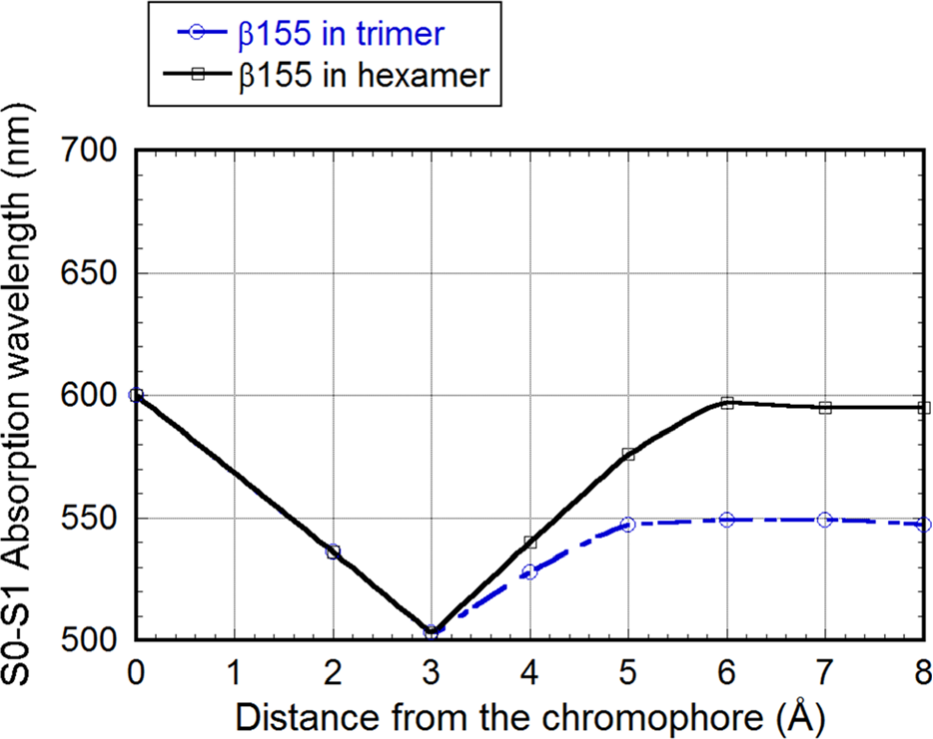
Dependence of λ_1_ for the S_0_→S_1_ transition on distance *R* from the β155 chromophore of the trimer and hexamer models, calculated by including the electrostatic interaction of β155 with protein moieties and water molecules within *R* Å.

Figure 5 shows the dependence of λ_1_ on distance *R* from β155 for the trimer and hexamer models; the surrounding atoms within *R* from β155 were incorporated in the calculation. When *R* is up to 3 Å, both the trimer and hexamer models are the same. However, when *R* ≥ 4 Å, the hexamer model shows the effect of the K chain. The λ_1_ value for β155 alone was 600 nm. Hence, it is clear that the short‐wavelength shift of the absorption wavelength is due to the effect of the protein moieties of the trimer and the water molecules surrounding β155. Conversely, the effect of the K chain, that is, the role of the hexamer, results into the long‐wavelength shift, which counteracts the effect of the protein moieties of the trimer and the water molecules surrounding β155. Additionally, when the K chain part is removed from the hexamer model (Fig. 4D), the λ_1_ and *f*_1_ of *R* = 7 Å and *R* = 8 Å are 547 nm and 1.36, or 546 nm and 1.47, respectively. These results show that the hexamer leads to the long‐wavelength shift.

## Discussion

The electronic state and light absorption properties of α84, β84, and β155 in C‐PC were calculated for both the trimer and hexamer models using the INDO‐CI method based on the crystal structure 1CPC. When the chromophore is electrically neutral, the oscillator strength *f*_1_ for the transition (S_0_→S_1_) from the ground state to the first excited state is small and does not agree with the experimental result [25, 26]. However, when the chromophore is protonated, *f*_1_ for the S_0_→S_1_ transition becomes large and agrees with the experimental results. Thus, the protonated form was used for the calculations, and λ_1_ (λ_max_), which is the absorption wavelength for the S_0_→S_1_ transition, was utilized as an index for the effect of the environment in this study. The protein moieties and water molecules within 8 Å from the chromophore were taken as the environmental effect and considered in the calculation.

In the hexamer model, the calculated absorption wavelengths of α84, β84, and β155 agreed with the experimental results for native PBS. In the case of the trimer model, in contrast, only the result of β155, which shifted to the shorter‐wavelength side, did not agree with the experiment. The absorption wavelength of β155 was ~ 550 nm for the trimer model, while it was ~ 595 nm for the hexamer model.

More light energy can be harvested using a larger number of chromophores. The chromophore added to the PBS, except for α84 and β84, must be located outside the rod because of the molecular structure of PBS; β155 located outside the rod plays a role in absorbing more light energy. If β155 is surrounded by water, as in the trimer model, its optical absorption wavelength will become shorter than that of the hexamer model, which reflects native PBS. If the energy transfer in PBS can be considered in terms of Förster’s mechanism, then the efficiency of the energy transfer can be determined using the overlap integral of the emission spectra of β155 and the absorption spectra of α84 or β84 [32, 33, 34].

Thus, if the absorption wavelength of β155 is not close to that of α84 or β84, the efficiency of energy transfer from β155 to α84 or β84 will be small, and the light energy absorbed by β155 will be in vain. Thus, the light energy absorbed by β155 cannot be used effectively if the trimer is structurally independent and exists in the rod as a basic unit for function. However, if two (αβ)_3_‐trimers are associated face‐to‐face with each other and form a hierarchical structure as a (αβ)_6_‐hexamer, the light energy absorbed by β155 can be effectively transferred to α84 or β84 and utilized. Hence, one of the roles of the hierarchical structure of (αβ)_6_‐hexamer can be revealed by calculating the absorption wavelength.

Mimuro *et al*. determined the maximum light absorption of each chromophore in C‐PC obtained from *Mastigocladus laminosus* (α84: 618 nm, β84: 625 nm, β155: 594 nm) [7]; this assignment was verified by Siebzehnrübl *et al*. (α84: 617 nm, β84: 622 nm, β155: 598 nm) [30, 31]. However, Niedzwiedzki *et al*. [35] recently pointed out that, experimentally, there is no consensus specifically on the absorption wavelength of β155. Eisenberg *et al*. [36] showed the maximum β155 absorption at ~ 550 nm, and Gryliuk *et al*. [37] showed that the 4 K absorption spectrum of PBS from *Acaryochloris marina* partially resolves two bands at 574 and 599 nm. This study might support these experimental results and may have implications for optical absorption experiments on the monomer, trimer, and hexamer in the PBS rod.

One might argue that the semi‐empirical MO method is insufficient. Indeed, it is impossible to obtain very high precision values using semi‐empirical MO methods; however, it is useful to use the obtained value as an index and for discussion. Herein, the parameters of the INDO‐CI method were set to reproduce the experimental values for the optical absorption wavelengths of α84, and these parameters were also applied to determine the optical absorption wavelengths of β84 and β155. Only β155 in the trimer model showed a shorter‐wavelength shift of ~ 40 nm. High precision values are not needed for this discussion. Namely, it is not too much to say that only the case of β155 in the trimer model is qualitatively different from the other cases, showing such a large blue shift, in which the value of the overlap integral in Förster's formula [32, 33, 34] becomes smaller than that of the hexamer model. Accordingly, this discussion based on the results of the calculations using the INDO‐CI method is effective and valuable.

If we consider one protein chain as the smallest unit of structure, PBS can interestingly be considered to be composed of several hierarchical structures with different functions. First, the α‐subunit or β‐subunit consists of a globinfold part and X‐Y helices [4, 5, 13]. The Asp87 of globinfolds promotes the protonation of the chromophore, which enables the light harvesting of ~ 620 nm [25, 26]. The monomer forms the second hierarchical structure. The X‐Y helix portion of the subunit associates the α‐subunit with the β‐subunit and also simultaneously prevents the Asp87 fluctuations from increasing so as to stabilize the protonation of the chromophore [38, 39]. The trimer forms the third hierarchical structure, which is a structural unit assembling PBS. The hexamer, the fourth hierarchical structure, modulates the optical absorption wavelength of β155 and effectively transfers the light energy to α84 or β84. The molecules that play a central role in the fifth hierarchical structure are thought to be linker proteins, which are closely related to the energy transfer in the PBS rod. Recently, the complete structure of PBS, including the linker proteins, has been clarified [40], which will lead to the discovery of new relationships between the PBS structure and function in the near future.

## Conflict of interest

The authors declare no conflict of interest.

## Author contributions

HK conceived the study, performed the calculations and the analyses, and wrote the manuscript.

## Data Availability

All data generated or analyzed during this study are included in this published article.

## References

[1] Gantt E (1981) Phycobilisomes. Ann Rev Plant Physiol 32, 327–347.

[2] Gantt E (1994) Supramolecular membrane organization In The Molecular Biology of Cyanobacteria (Bryant DA, ed), pp. 119–138. Springer, Dordrecht.

[3] Sidler WA (1994) Phycobilisomes and phycobiliprotein structures In The Molecular Biology of Cyanobacteria (Bryant DA, ed), pp. 139–216. Springer, Dordrecht.

[4] Schirmer T , Bode W , Huber R , Sidler WA and Zuber H (1985) X‐ray crystallographic structure of the light‐harvesting biliprotein C‐phycocyanin from the thermophilic cyanobacterium *Mastigocladus laminosus* and its resemblance to globin structures. J Mol Biol 184, 257–277. 3928897 10.1016/0022-2836(85)90379-1

[5] Schirmer T , Huber R , Schneider M , Bode W , Miller M and Hackert ML (1986) Crystal structure analysis and refinement at 2·5 Å of hexameric C‐phycocyanin from the cyanobacterium *Agmenellum quadruplicatum*: the molecular model and its implications for light‐harvesting. J Mol Biol 188, 651–676. 3090271 10.1016/s0022-2836(86)80013-4

[6] David L , Marx A and Adir N (2011) High‐resolution crystal structures of trimeric and rod phycocyanin. J Mol Biol 405, 201–213. 21035460 10.1016/j.jmb.2010.10.036

[7] Mimuro M , Füglistaller P , Rümbeli R and Zuber H (1986) Functional assignment of chromophores and energy transfer in C phycocyanin isolated from the thermophilic cyanobacterium *Mastigocladus laminosus* . Biochim Biophys Acta Bioenerg 848, 155–166.

[8] Chenu A , Keren N , Paltiel Y , Nevo R , Reich Z and Cao J (2017) Light adaptation in phycobilisome antennas: influence on the rod length and structural arrangement. J Phys Chem B 121, 9196–9202. 28872312 10.1021/acs.jpcb.7b07781

[9] Herrera‐Salgado P , Leyva‐Castillo LE , Rios‐Castro E and Gómez‐Lojero C (2018) Complementary chromatic and far‐red photoacclimations in *Synechococcus* ATCC 29403 (PCC 7335). I: the phycobilisomes, a proteomic approach. Photosynth Res 138, 39–56. 29943359 10.1007/s11120-018-0536-6

[10] Hirose Y , Chihong S , Watanabe M , Yonekawa C , Murata K , Ikeuchi M and Eki T (2019) Diverse chromatic acclimation processes regulating phycoerythrocyanin and rod‐shaped phycobilisome in cyanobacteria. Int J Biol Macromol 137, 647–656.31265852

[11] Green BR (2019) What happened to the Phycobilisome? Biomolecules 9, 748–758. 10.3390/biom9110748 PMC6921069 31752285

[12] Adir N , Bar‐Zvi S and Harris D (2020) The amazing phycobilisome. Biochim Biophys Acta Bioenerg 1861, 148047. 31306623 10.1016/j.bbabio.2019.07.002

[13] Dürring M , Schmidt GB and Huber R (1991) Isolation, crystallization, crystal structure analysis and refinement of constitutive C‐phycocyanin from the chromatically adapting cyanobacterium *Fremyella diplosiphon* at 1.66 Å resolution. J Mol Biol 217, 577–592. 1899708 10.1016/0022-2836(91)90759-y

[14] Stewart JJP (1989) Optimization of parameters for semi‐empirical methods I‐method. J Comp Chem 10, 209–220.

[15] Stewart JJP (1990) MOPAC: a semiempirical molecular orbital program. J Comput Aided Mol Des 4, 1–103. 2197373 10.1007/BF00128336

[16] Salomon‐Ferrer R , Case DA and Walker RC (2013) An overview of the Amber biomolecular simulation package. WIREs Comput Mol Sci 3, 198–210.

[17] Duan Y , Wu C , Chowdhury S , Lee MC , Xiong G , Zhang W , Yang R , Cieplak P , Luo R , Lee T *et al* (2003) A point‐charge force field for molecular mechanics simulations of proteins based on condensed‐phase quantum mechanical calculations. J Comput Chem 24, 1999–2012. 14531054 10.1002/jcc.10349

[18] Lee MC and Duan Y (2004) Distinguish protein decoys by using a scoring function based on a new AMBER force field, short molecular dynamics simulations, and the gereralized born solvent model. Proteins 55, 620–634. 15103626 10.1002/prot.10470

[19] Berendsen HJC , Postma JPM , van Gunsteren WF , Dinola A and Haak JR (1984) Molecular dynamics with coupling to an external bath. J Chem Phys 81, 3684.

[20] Darden T , York D and Pedersen L (1993) Particle mesh Ewald: an N·log(N) method for Ewald sums in large systems. J Chem Phys 98, 10089–10092.

[21] Frisch MJ , Trucks GW , Schlegel HB , Scuseria GE , Robb MA , Cheeseman JR , Scalmani G , Barone V , Mennucci B , Petersson GA *et al* (2009) Gaussian 09, Revision A. 02, Gaussian Inc., Connecticut https://gaussian.com/

[22] Sakuranaga M , Nakachi K and Suzuki H (1979) Theory of formamide and its related compounds by INDO‐CI method. J Phys Soc Jpn 46, 944–951.

[23] Pople JA , Beveridge DL and Dobosh PA (1967) Approximate self‐consistent molecular‐orbital theory. V. Intermediate neglect of differential overlap. J Chem Phys 47, 2026–2033.

[24] Wolfsberg M and Helmholtz L (1952) The spectra and electronic structure of the tetrahedral ions MnO_4_ ^−^, CrO_4_ ^−−^, and ClO_4_ ^−^ . J Chem Phys 20, 837–843.

[25] Kikuchi H , Sugimoto T and Mimuro M (1997) An electronic state of the chromophore, phycocyanobilin, and its interaction with the protein moiety in C‐phycocyanine: protonation of the chromophore. Chem Phys Lett 274, 460–465.

[26] Mimuro M , Kikuchi H and Murakami A (1999) Structure and Function of Phycobilisomes In Concepts in Photobiology: Photosynthesis and Photomorphogenesis (Singhal GS, Renger G, Sopory SK, Irrgang KD and Govindjee eds), pp. 104–135. Springer, Dordrecht.

[27] Kneip C , Hildebrandt P , Mark F and Schaffner K (1999) Interpretation of the resonance Raman spectra of linear tetrapyrroles based on DFT calculations. Chem Phys Lett 311, 479–484.

[28] Mroginski MA , Mark F , Thiel W and Hildebrandt P (2007) Quantum mechanics/molecular mechanics calculation of the Raman Spectra of the Phycocyanobilin Chromophore in alpha‐C‐phycocyanin. Biophys J 93, 1885–1894. 17513350 10.1529/biophysj.107.108878 PMC1959556

[29] Corbella M , Toa ZSD , Scholes GD , Luque FJ and Curutchet C (2018) Determination of the protonation preferences of bilin pigments in cryptophyte antenna complexes. Phys Chem Chem Phys 20, 21404–21416. 30105318 10.1039/c8cp02541j

[30] Siebzehnrübl S , Fischer R and Scheer H (1987) Chromophore assignment in phycocyanin from *Mastigocladus laminosus* . Z Naturforsch C 42, 258–262.

[31] Sauer K , Scheer H and Sauer P (1987) Förster transfer calculations based on crystal structure data from *Agmenellum quadruplicatum* C‐phycocyanin. Photochem Photobiol 46, 427–440.

[32] Förster T (1948) Zwischenmolekulare Energiewanderung und Fluoreszenz. Ann Physik 2, 55–75.

[33] Förster T . (1967) Mechanisms of energy transfer In Comprehensive Biochemistry (Florkin M and Stotz EH eds). Vol 22, pp. 61–80. Elsevier, Amsterdam.

[34] Feron E , Belcher WJ , Fell CJ and Dastoor PC (2012) Organic solar cells: understanding the role ot Förster resonance energy transfer. Int J Mol Sci 13, 17019–17047. 23235328 10.3390/ijms131217019 PMC3546737

[35] Niedzwiedzki DM , Bar‐Zvi S , Blankenship RE and Adir N (2019) Mapping the excitation energy migration pathways in phycobilisomes from the cyanobacterium *Acaryochloris marina* . Biochim Biophys Acta Bioeneg 1860, 286–296. 10.1016/j.bbabio.2019.01.002 30703363

[36] Eisenberg I , Caycedo‐Soler F , Harris D , Yochelis S , Huelga SF , Plenio MB , Adir N , Keren N and Paltiel Y (2017) Regulating the energy flow in a cyanobacterial light‐harvesting antenna complex. J Phys Chem B 121, 1240–1247. 28121148 10.1021/acs.jpcb.6b10590

[37] Gryliuk G , Ratsep M , Hildebrandt S , Irrgang KD , Eckert HJ and Pieper J (2014) Excitation energy transfer and electron‐vibrational coupling in phycobiliproteins of the cyanobacterium *Acaryochloris marina* investigated by site‐selective spectroscopy. Biochim Biophys Acta Bioenerg 1837, 1490–1499. 10.1016/j.bbabio.2014.02.010 24560813

[38] Kikuchi H , Wako H , Yura K , Go M and Mimuro M (2000) Significance of a two‐domain structure in subunits of phycobiliproteins revealed by the normal mode analysis. Biophys J 79, 1587–1600. 10969019 10.1016/S0006-3495(00)76409-5 PMC1301051

[39] Mimuro M and Kikuchi H (2003) Antenna systems and energy transfer in Cyanophyta and Rhodophyta In Light‐Harvesting Antennas in Photosynthesis. Advances in Photosynthesis and Respiration (Green BR and Parson WW eds). Vol 13, pp. 281–306. Kluwer Academic Publishers, Dordrecht/Boston/London.

[40] Ma J , You X , Sun S , Wang X , Qin S and Sui SF (2020) Structural basis of energy transfer in *Porphyridium purpureum* phycobilisome. Nature 579, 146–151. 32076272 10.1038/s41586-020-2020-7

